# Time course study of oxidative and nitrosative stress and antioxidant enzymes in K_2_Cr_2_O_7_-induced nephrotoxicity

**DOI:** 10.1186/1471-2369-6-4

**Published:** 2005-04-26

**Authors:** José Pedraza-Chaverrí, Diana Barrera, Omar N Medina-Campos, Raymundo C Carvajal, Rogelio Hernández-Pando, Norma A Macías-Ruvalcaba, Perla D Maldonado, Marcos I Salcedo, Edilia Tapia, Liliana Saldívar, María E Castilla, María E Ibarra-Rubio

**Affiliations:** 1 Facultad de Química, Departamento de Biología, Edificio B, Segundo Piso, Laboratorio 209, Universidad Nacional Autónoma de México (UNAM), Ciudad Universitaria, 04510, México, D.F., México; 2 Facultad de Medicina, Departamento de Farmacología, Universidad Nacional Autónoma de México (UNAM), Ciudad Universitaria, 04510, México, D.F., México; 3 Instituto Nacional de Ciencias Médicas y Nutrición "Salvador Zubirán", Departamento de Patología, 14000, México, D.F., México; 4 Facultad de Química, Edificio B, Laboratorio 124, Departamento de Química Orgánica, Universidad Nacional Autónoma de México (UNAM), Ciudad Universitaria 04510, México, D.F., México; 5 Departamento de Nefrología, Instituto Nacional de Cardiología "Ignacio Chávez", Juan Badiano #1, Col Sección XVI, 14080 Tlalpan, México, D.F., México; 6 Facultad de Química, Edificio B, Departamento de Química Analítica, Universidad Nacional Autónoma de México (UNAM), Ciudad Universitaria 04510, México, D.F., México

## Abstract

**Background:**

Potassium dichromate (K_2_Cr_2_O_7_)-induced nephrotoxicity is associated with oxidative and nitrosative stress. In this study we investigated the relation between the time course of the oxidative and nitrosative stress with kidney damage and alterations in the following antioxidant enzymes: Cu, Zn superoxide dismutase (Cu, Zn-SOD), Mn-SOD, glutathione peroxidase (GPx), glutathione reductase (GR), and catalase (CAT).

**Methods:**

Nephrotoxicity was induced in rats by a single injection of K_2_Cr_2_O_7_. Groups of animals were sacrificed on days 1,2,3,4,6,8,10, and 12. Nephrotoxicity was evaluated by histological studies and by measuring creatinine clearance, serum creatinine, blood urea nitrogen (BUN), and urinary excretion of N-acetyl-β-D-glucosaminidase (NAG) and total protein. Oxidative and nitrosative stress were measured by immunohistochemical localization of protein carbonyls and 3-nitrotyrosine, respectively. Cu, Zn-SOD, Mn-SOD, and CAT were studied by immunohistochemical localization. The activity of total SOD, CAT, GPx, and GR was also measured as well as serum and kidney content of chromium and urinary excretion of NO_2 _^-^/NO_3_^-^. Data were compared by two-way analysis of variance followed by a post hoc test.

**Results:**

Serum and kidney chromium content increased reaching the highest value on day 1. Nephrotoxicity was made evident by the decrease in creatinine clearance (days 1–4) and by the increase in serum creatinine (days 1–4), BUN (days 1–6), urinary excretion of NAG (days 1–4), and total protein (day 1–6) and by the structural damage to the proximal tubules (days 1–6). Oxidative and nitrosative stress were clearly evident on days 1–8. Urinary excretion of NO_2_^-^/NO_3_^- ^decreased on days 2–6. Mn-SOD and Cu, Zn-SOD, estimated by immunohistochemistry, and total SOD activity remained unchanged. Activity of GPx decreased on days 3–12 and those of GR and CAT on days 2–10. Similar findings were observed by immunohistochemistry of CAT.

**Conclusion:**

These data show the association between oxidative and nitrosative stress with functional and structural renal damage induced by K_2_Cr_2_O_7_. Renal antioxidant enzymes were regulated differentially and were not closely associated with oxidative or nitrosative stress or with kidney damage. In addition, the decrease in the urinary excretion of NO_2_^-^/NO_3_^- ^was associated with the renal nitrosative stress suggesting that nitric oxide was derived to the formation of reactive nitrogen species involved in protein nitration.

## Background

K_2_Cr_2_O_7 _is a chemical compound that is widely used in metallurgy, chrome plating, chemical industry, textile manufacture, wood preservation, photography and photoengraving, refractory and stainless steel industry, and cooling systems [1]. Occupational exposure to chromium has been associated with welders, chrome-plating workers, and chromium pigment factories workers [2]. Chromium is known to cause allergic dermatitis [3], carcinogenicity [4], and ARF in humans [2,5,6] and in animals [7-11].

Acute renal failure (ARF) induced by potassium dichromate (K_2_Cr_2_O_7_) has been used as a model to study the pathophysiology of this disease [7,9]. Experimental data show that chromate affects selectively the convoluted section of the proximal tubules [7,12-15] and induces acute necrosis of renal tubules [14,15]. Clinical and experimental renal damage induced by K_2_Cr_2_O_7 _has been associated with oxidative stress [15-20]. In fact, some antioxidants such as ascorbic acid, vitamin E, N-acetyl cysteine, and glutathione prevent the K_2_Cr_2_O_7 _induced renal damage [21-25] whereas the inhibition of glutathione biosynthesis enhances it [22,24]. Evidences suggest that reactive oxygen species (ROS) are involved in Cr(VI)-induced cell injury [16,17,19,26]. Chromium reduction intermediates [Cr(V), Cr(IV), and Cr(III)], which could be generated under physiological conditions, may be toxic as they involve ROS production [27-29]. *In vitro *chromate reduction via H_2_O_2 _has been shown to produce hydroxyl radicals via a Fenton-like reaction [27,30-33].

We recently showed that stannous chloride (SnCl_2_) pretreatment has a protective role in K_2_Cr_2_O_7_-induced nephrotoxicity [15,34]. SnCl_2 _is a potent inductor of heme oxygenase-1 [15,35] and the protective role of SnCl_2 _in this experimental model has been attributed, at least in part, to the heme oxygenase-1 preinduction [15,34]. We also showed that renal activity of Cu, Zn-SOD, Mn-SOD, and GR remained unchanged at 24 and 48 h whereas GPx and CAT activities remained unchanged at 24 h but decreased at 48 h in K_2_Cr_2_O_7_-injected rats. In the present work we performed a time course study of functional and structural renal damage, oxidative and nitrosative stress, and the behavior of the antioxidant enzymes Cu, Zn superoxide dismutase (Cu, Zn-SOD), Mn superoxide dismutase (Mn-SOD), glutathione reductase (GR), glutathione peroxidase (GPx), and catalase (CAT) in K_2_Cr_2_O_7_-induced nephrotoxicity to know if the antioxidant enzymes respond in a coordinate way and if there is an association between (a) oxidative/nitrosative stress and renal damage, (b) oxidative/nitrosative stress and the antioxidant enzymes, and (c) antioxidant enzymes and renal damage.

## Methods

### Reagents

Formaldehyde, anhydrous absolute ethanol, xylol, methanol, chloroform, and K_2_Cr_2_O_7 _were from J.T. Baker (México, D.F.), p-nitrophenyl-N-acetyl-β-D-glucosaminide, acrylamide, NADPH, N, N'-methylene-bis-acrylamide, xanthine, xanthine oxidase, nitroblue tetrazolium (NBT), β-nicotinamide adenine dinucleotide phosphate, reduced form (β-NADPH), aprotinin, leupeptin, pepstatin, and 2,4-dinitrophenylhydrazine (DNPH) were from Sigma Chemical Co. (St. Louis, MO, USA). Commercial kits to measure creatinine and blood urea nitrogen were from Spinreact (Girona, Spain). Rabbit anti-rat polyclonal antibodies against Mn-SOD (Cat. # SOD 111) and Cu, Zn-SOD (Cat. # SOD 101) were from Stressgen Biotechnologies Co. (Victoria, BC, Canada). Rabbit anti-human CAT polyclonal antibodies (Cat. # 219010) were from Calbiochem (San Diego, CA, USA). Goat anti-dinitrophenol (DNP) polyclonal antibodies (Cat. # J06) were from Biomeda Corporation (Foster City, CA, USA). Rabbit anti-3-nitrotyrosine (3-NT) polyclonal antibodies (Cat. #06-284) were from Upstate (Lake Placid, NY, USA). Anti-rabbit immunoglobulin horseradish peroxidase antibodies (Cat. # NA-934) were from Amersham (Buckinghamshire, UK). Donkey anti-goat horseradish peroxidase antibodies (Cat. # SC2020) were from Santa Cruz Biotechnology, Inc. (Santa Cruz, CA, USA). Enhanced chemiluminiscence (ECL) kit for Western blot was purchased from Amersham Life Sciences (Buckinghamshire, England). All other chemicals were reagent grade and commercially available.

### Experimental design

Seven-week-old male Wistar rats with an initial body weight of 200–210 g were used. Experimental work was approved by CONACYT (#25441) and DGAPA (IN210201) and followed the guidelines of Norma Oficial Mexicana (NOM-ECOL-087-1995). Two groups of rats were studied: 1) CT, control injected subcutaneously with 0.5 ml isotonic saline solution (n = 40); and 2) K_2_Cr_2_O_7_, treated with a single subcutaneous injection of 15 mg/Kg K_2_Cr_2_O_7 _[7] in a volume of 0.5 ml (n = 43). The study was performed in two stages: rats from days 1,2,3,4, and 6 were studied in the first one (n = 5/per group) and rats from days 8, 10, and 12 were studied in the second one (n = 5 for control group and n = 6 for K_2_Cr_2_O_7 _group). Rats were sacrificed on days 1,2,3,4,6,8,10, and 12. There was no mortality. Rats had free access to water and CT group was pair-fed to match the food intake of K_2_Cr_2_O_7 _group. Animals were maintained in metabolic cages to collect 24-h urine to measure N-acetyl-β-D-glucosaminidase (NAG), total protein, creatinine, and NO_2_^-^/NO_3_^-^.

Animals were sacrificed by decapitation and trunk blood was collected at room temperature to obtain serum to measure chromium content and the markers of renal function, creatinine and blood urea nitrogen (BUN). Glomerular filtration rate was estimated by creatinine clearance. The kidneys were obtained to perform histological analysis, chromium determination, the activity of antioxidant enzymes, Western blot of CAT, Cu, Zn-SOD, and Mn-SOD, and the immunohistochemical localization of 3-NT, protein carbonyls, CAT, Cu, Zn-SOD, and Mn-SOD.

### Chromium concentration

Chromium content in serum and in total kidney was measured by graphite-furnace atomic-absorption spectrometry on a Perkin Elmer 3110 device with furnace.

### Markers of nephrotoxicity

Creatinine and BUN were measured using commercial kits according to the instructions of manufacturers and were expressed as mg/dL. Creatinine clearance was calculated with the serum and urine data according to the standard formula. Urinary NAG excretion was determined at 405 nm using p-nitrophenyl-N-acetyl-β-D-glucosaminide as substrate and the data were expressed as U/24 h [7,15]. One unit of NAG was defined as the amount of enzyme that releases 1 μmol of p-nitrophenol in the assay conditions. Total protein in urine was measured by a turbidimetric method with 12.5% trichloroacetic acid at 420 nm [7,15] and the data were expressed as mg/24 h.

### Histological studies

Thin slices of kidney tissue with cortex and medulla were fixed by immersion in buffered formalin (pH 7.4), dehydrated and embedded in paraffin. Sections (4 μm) were stained with hematoxylin and eosin (H&E) [15]. A quantitative histological damage was determined by using a Leica Qwin Image Analyzer (Cambridge, UK). The histological profile of twenty proximal tubules randomly selected per rat (5 rats per group) was recorded (n = 100 tubuli/group at each time point). The number of tubules with histopathological alterations like swelling, cytoplasmic vacuolization, desquamation or necrosis was registered and the data were expressed as percentage of damaged tubules. The percentage of damaged tubules of K_2_Cr_2_O_7 _and control groups was compared.

### Immunohistochemical localization of 3-NT, protein carbonyls, Cu, Zn-SOD, Mn-SOD, and CAT

For immunohistochemistry, 4 μm sections were deparaffined with xylol and rehydrated with ethanol. Endogenous peroxidase was quenched/inhibited with 4.5% H_2_O_2 _in methanol by incubation for 1.5 h at room temperature. The sections used for DNP immunohistochemistry were incubated with 0.2% DNPH in 2 N HCl for 60 min at room temperature in absence of light and then were extensively washed with phosphate buffer saline. Nonspecific adsorption was minimized by leaving the sections in 3% bovine serum albumin in phosphate buffer saline for 30 min. Sections were incubated overnight with a 1:700 dilution of anti-3-NT antibodies [15], or a 1:500 dilution of anti-DNP antibodies, or a 1:250 dilution of anti Cu, Zn-SOD antibodies, or a 1:500 dilution of anti Mn-SOD antibodies, or a 1:500 dilution of anti CAT antibodies. The sections were incubated with a 1:500 dilution of a peroxidase conjugated anti-rabbit immunoglobulin antibodies (for 3-NT, Cu, Zn-SOD, Mn-SOD, and CAT) or with a 1:500 dilution of a peroxidase conjugated anti-goat Ig (for DNP) for 1 h, and finally incubated with H_2_O_2_-diaminobenzidine for 1 min. Sections were counterstained with hematoxylin and observed under light microscopy. All the sections were incubated under the same conditions with the same concentration of antibodies, and in the same running, so the immunostaining was comparable among the different experimental groups. The stained area was quantified using a SigmaScan Pro (version 4.01.003) (Jandel Scientific, San Rafael, CA). The data are expressed as percent respect to day 0.

### Urinary excretion of NO_2_^-^/NO_3_^-^

Nitric oxide (NO) is a labile substance with a short half-life and it decomposes rapidly to NO_2_^- ^and NO_3_^- ^in biological solutions [36], and these stable breakdown products have been measured as an index of NO production [37]. NO_2_^- ^and NO_3_^- ^were measured in the 24-hour urine samples at all time points. Urine samples were first incubated with *E. coli *nitrate reductase to convert the NO_3_^- ^to NO_2_^-^, as described previously [38,39]. To prepare this enzyme, *E. coli *was grown for 18 hours under anaerobic conditions in nitrate rich medium, washed, resuspended in phosphate buffer and frozen at -70°C until use. The samples were incubated with the enzyme in phosphate ammonium formate buffer (pH 7.3) for one hour at 37°C. After incubation, total NO_2_^- ^in the samples (representing both NO_2_^- ^and reduced NO_3_^-^) was measured using the Griess reagent. Known concentrations of NaNO_2 _and NaNO_3 _were used as standards in each assay. Data were expressed as nmol/min.

### Activity of antioxidant enzymes

Kidney was homogenized in a Polytron (Model PT 2000, Brinkmann, Westbury, NY, USA) for 10 seconds in cold 50 mM potassium phosphate, 0.1% Triton X-100, pH = 7.0. The homogenate was centrifuged at 19,000 × g and 4°C for 30 min and the supernatant was separated to measure total protein and the activities of CAT, GPx, GR, and total SOD.

Total SOD activity in renal cortex homogenates was assayed spectrophotometrically at 560 nm by a previously reported method using NBT as the indicator reagent [34]. The amount of protein that inhibited NBT reduction to 50% of maximum was defined as one unit of SOD activity. Results were expressed as U/mg protein. CAT activity in renal cortex, was assayed by a method based on the disappearance of 30 mM H_2_O_2 _at 240 nm [34] and the data were expressed as *k*/mg protein. GPx activity in renal cortex was measured at 340 nm using GR and NADPH in a coupled reaction [34]. One unit of GPx was defined as the amount of enzyme that oxidize 1 μmol of NADPH/min. Data were expressed as U/mg protein. GR activity in renal cortex was assayed using oxidized glutathione as substrate and measuring the disappearance of NADPH at 340 nm [34]. One unit of GR was defined as the amount of enzyme that oxidize 1 μmol of NADPH/min. Data were expressed as U/mg protein.

### Western blot

Renal cortex (100 mg) was homogenized in 300 μl of phosphate buffer 50 mM, pH 7.4 containing the following cocktail of protease inhibitors: leupeptin 5 μg/ml, pepstatin 7 μg/ml, aprotinin 5 μg/ml, and EDTA 1 mM. The homogenates were centrifuged at 1,000 × g and 4°C for 10 min. Thirty μg of protein were fractionated by reducing 12.5% sodium dodecyl sulfate polyacrylamide gel electrophoresis and electroblotted to a nitrocellulose membrane (Hybond™ ECL™, Amersham, Buckinghamshire, England). Immunodetection was performed using specific primary antibodies against CAT (1:500 dilution), Mn-SOD (1,5000 dilution), or Cu, Zn-SOD (1:5,000 dilution). Membranes were then probed with the appropriate secondary antibody-peroxidase conjugate (1:5,000 dilution). The hybrids were visualized using ECL detection system and quantified by densitometry (Sigma ScanPro, version 4.0, San Rafael, CA, USA). Data were expressed as % of the control.

### Statistics

The data are expressed as the mean ± SEM and were analyzed by two-way analysis of variance followed by Bonferroni t-test using the software Prism 2.01 (GraphPad, San Diego, CA, USA). A P value less than 0.05 was considered statistically significant.

## Results and discussion

**Figure 1:**
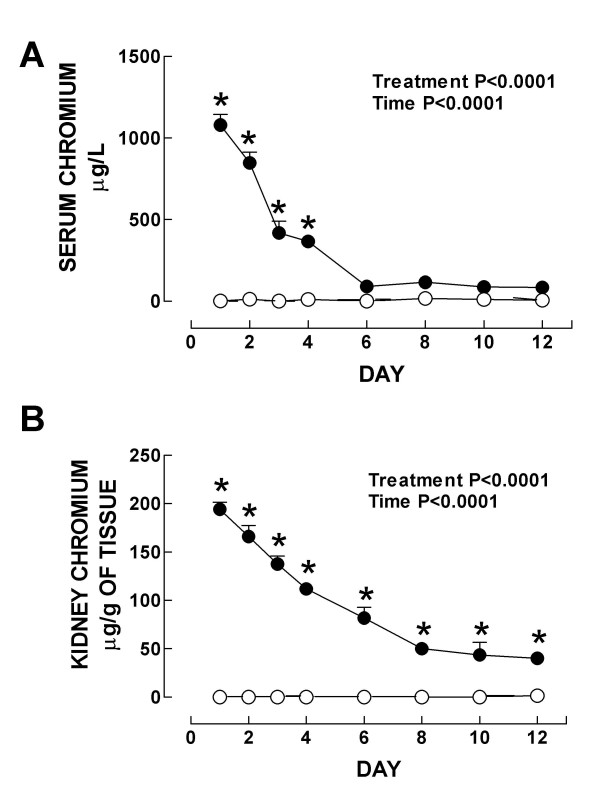
Chromium concentration in (A) serum and (B) kidney in control (○) and K_2_Cr_2_O_7 _(●)-treated rats. Data are mean ± SEM. *P < 0.001 vs. control group. *n *= 3–6.

ROS have been involved in the pathophysiology of ARF induced by K_2_Cr_2_O_7 _and some antioxidants are able to ameliorate renal damage induced by this compound [15,16,18,23,25]. In addition, it has been previously shown that renal heme oxygenase-1 preinduction by SnCl_2 _ameliorates ARF and prevents oxidative and nitrosative stress induced 24 h after K_2_Cr_2_O_7 _injection [15,34]. In the present work we performed a time-course analysis of the nephrotoxicity, oxidative and nitrosative stress, and changes in antioxidant enzymes induced by K_2_Cr_2_O_7_. Body weight of control and K_2_Cr_2_O_7_-treated rats was similar at all time points (see additional file 1). Urinary volume was increased in K_2_Cr_2_O_7_-treated animals on days 3 (1.9-fold) and 4 (1.6-fold) and returned to control values thereafter (see additional file 1). Polyuria observed in our rats is consistent with previous data [2]. We measured the chromium content in serum and kidney and it was found that it increased on days 1–6 and 1–12, respectively (Fig. 1). In both cases the peak value was reached on day 1 and then the chromium concentration decreased gradually, but in kidney remained still significantly high on day 12 and in serum reached values not different from control rats since day 6. Our data are consistent with previous pharmacokinetic studies which have shown that chromium is rapidly distributed [40] and that the half life of chromium is longer in kidney than in blood serum [41].

**Figure 2:**
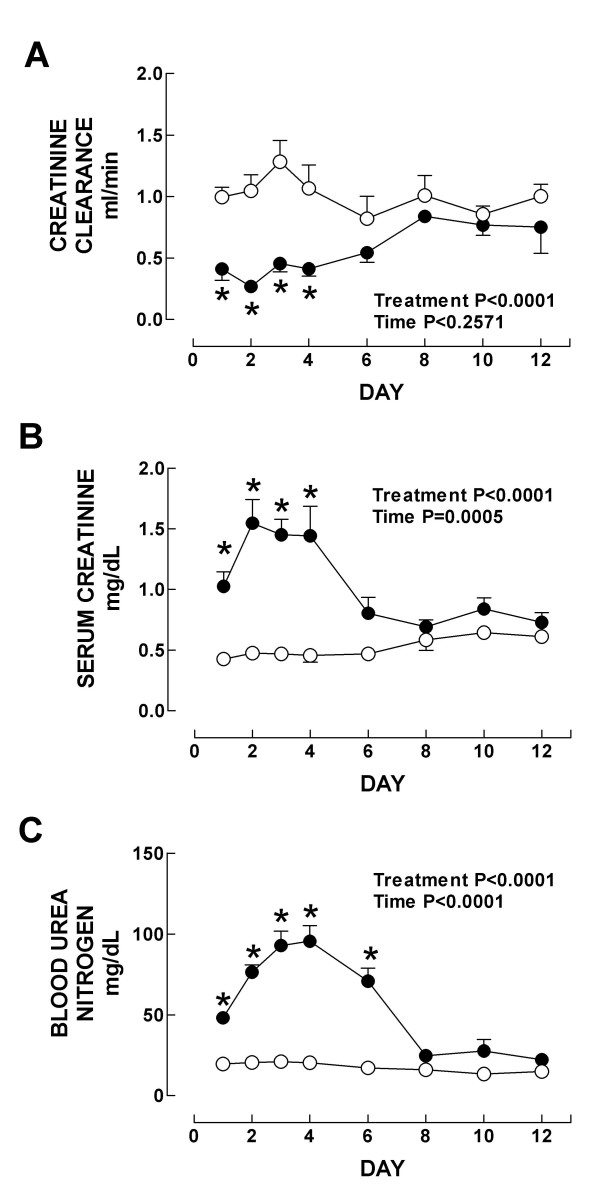
(A) Creatinine clearance, (B) serum creatinine, and (C) blood urea nitrogen control (○) and K_2_Cr_2_O_7 _(●)-treated rats. Data are mean ± SEM. *P at least <0.05 vs. control group. *n *= 4–6.

**Figure 3:**
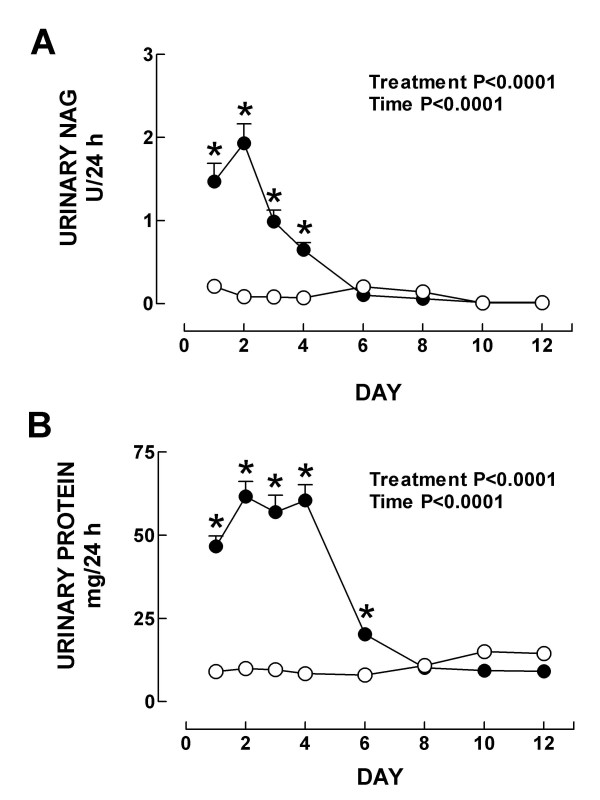
Urinary excretion of (A) NAG and (B) total protein in control (○) and K_2_Cr_2_O_7 _(●)-treated rats. Data are mean ± SEM. *P at least <0.05 vs. control group. *n *= 4–18.

**Figure 4:**
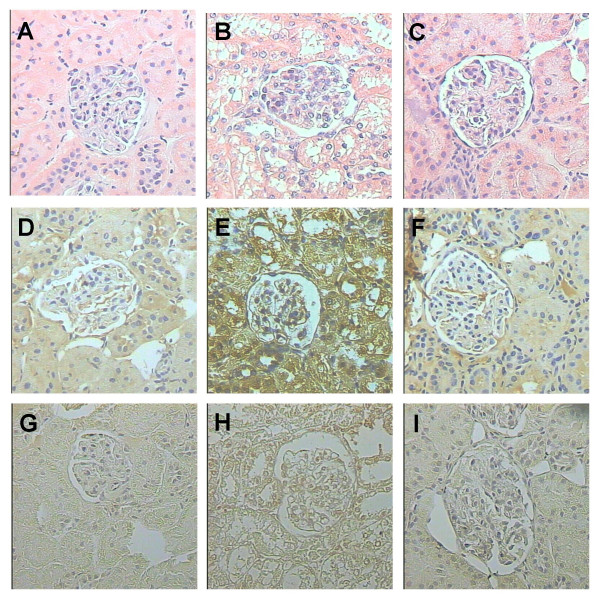
Representative images of histology (first row) and immunohistochemical detection of 3-NT (second row) and DNP (third row) during the evolution of the kidney damage produced by K_2_Cr_2_O_7_. The study was performed in control rats (day 0) and on days 2 and 12 after a single injection of K_2_Cr_2_O_7 _(15 mg/Kg). (A) Normal kidney histology from control rat (H/E). (B) On day 2, there is extensive tubular damage manifested by swollen and necrotic epithelial cells (H/E). (C) total kidney regeneration has been produced on day 12 post K_2_Cr_2_O_7 _administration (H/E). (D) Normal kidney from control animals shows scarce 3-NT immunoreactivity. (E) On day 2, there is strong 3-NT immunostaining in the epithelium from the convoluted tubules. (F) Slight 3-NT immunostaining is observed in the tubular epithelium on day 12. (G) Normal kidney from control rat shows scarce DNP immunostaining in the tubular epithelium. (H) In contrast, on day 2 there is clear DNP immunostaining in the cytoplasm and nucleus of the tubular epithelial cells and mesangium. (I) Slight DNP immunostaining is observed on day 12. 400 X.

**Figure 5:**
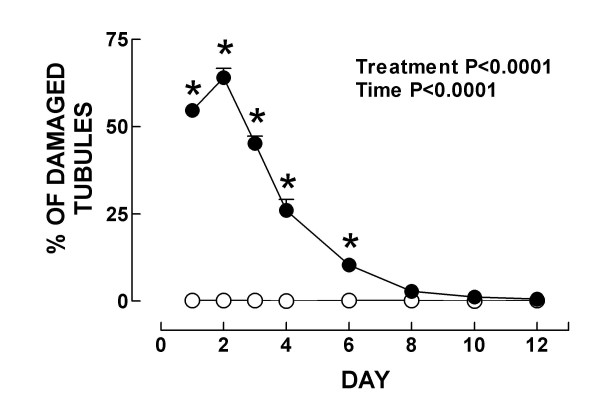
Quantitative histological analysis in control (○) and K_2_Cr_2_O_7 _(●)-treated rats. Data are mean ± SEM. *P < 0.05 vs. control group. *n *= 20 tubules/rat and 5 rats/group.

Markers of nephrotoxicity are shown in Figs. 2 and 3. Creatinine clearance decreased on days 1–4 (Fig. 2A), and serum creatinine and BUN increased on days 1–4 and 1–6, respectively (Figs. 2B and 2C). Urinary excretion of NAG and total protein increased on days 1–4 and 1–6, respectively (Figs. 3A and 3B). The major damage was observed on days 2–4 (Figs. 2 and 3). On day 8, all these markers returned to control values indicating that K_2_Cr_2_O_7_-induced ARF is reversible which is consistent with previous observations [10]. These functional findings are in a close agreement with the histological data. Significant structural abnormalities were seen in the kidney cortex since the first day after K_2_Cr_2_O_7 _administration. Specifically, 50% of the epithelium from the proximal convoluted tubules showed cellular swelling, necrosis and partial or complete detachment from the basal membrane. On day 2, when the peak of the tubular damage was observed, 70% of the proximal convoluted tubules was affected (Figs. 4B and 5). On days 3 and 4 proximal convoluted tubules showing the above mentioned abnormalities decreased to 40 and 25%, respectively (Fig. 5). On day 4, many tubules also showed numerous cellular debris in their lumen and epithelium regeneration was also seen but it was more evident on days 6 and 8 (data not shown). On days 10 and 12 the kidney histology was almost normal (Fig. 4C), only few tubules were revisted by active regenerative epithelium (small cuboidal cells with big nucleus and occasional mitotic figures). The high levels of serum and kidney chromium correlated with the structural and functional renal alterations on days 1–6. Interestingly, the renal damage disappeared in spite of the kidney still having high levels of chromium on days 8–12. This may be explained by the fact that chromium (VI), which is readily taken up into tissues, is reduced inside the cell to the final stable product chromium (III) [19]. The biological effects of chromium (VI) are generally attributed to cellular uptake, because chromium (VI), in contrast to chromium (III), is easily taken up by cells through the sulfate anion transport system [42,43]. However, once inside, chromium (VI) is reduced through reactive intermediates such as chromium (V) and chromium (IV) to the more stable chromium (III) by cellular reductants including glutathione, vitamins C and B_2_, and flavoenzymes [42]. Thus, the formation of chromium (III) or other intermediate oxidation states, in particular chromium (V), is believed to play a role in the biological effect of chromium (VI) compounds. *In vitro *studies have shown that this reduction process causes the generation of active oxygen species [44] which are involved in renal damage [15,34]. Interestingly, it has been shown that a low dose of K_2_Cr_2_O_7 _(10 mg/Kg) is unable to induce nephrotoxicity suggesting a threshold of this compound to induce renal damage [10]. In addition, it is know that chromium is located in vacuoles inside the proximal tubular cells which may delay the excretion of this metal [13]. In fact it has been shown that chromium remains for a long time in several tissues including kidney [13,40,41] which is consistent with our data.

The histological abnormalities correlated with renal functional alterations (days 1–6) and with the immunohistochemical detection of 3-NT and protein carbonyls. DNPH reacts with free carbonyls forming DNP-derived proteins which can be detected using antibodies against DNP. Carbonyl derivatives are formed by ROS mediated oxidation of side-chains of some amino acid residues and are important detectable markers of oxidative damage to proteins [45]. Kidneys from control non-treated animals did not show or only had slight immunostaining to 3-NT or protein carbonyls (Figs. 4D and 4G), while kidneys from K_2_Cr_2_O_7_-treated animals on day 2 showed strong 3-NT immunostaining in necrotic and swollen tubular epithelial cells, as well as some mesangial cells (Fig. 4E). This increase was significative (P < 0.0001 vs. day 0) (Table 1). In fact, it was observed from day 1 to day 8 (data not shown). Protein carbonyls immunostaining was also clear in the cytoplasm and nuclei from tubular epithelium and mesangial glomerular cells at the same time points (Fig. 4H and data not shown) (P < 0.001 vs. day 0) (Table 1). Then, on days 10 to 12, a striking decrease of 3-NT and DNP immunostaining was seen, being similar to the control non-treated rats (Figs. 4F and 4I). In previous works, we also have found in K_2_Cr_2_O_7_-treated rats an intense 3-NT immunostaining [15] and an increase in protein carbonyl content [15,34]. Our data confirm that ROS are involved in K_2_Cr_2_O_7_-induced nephrotoxicity [16,17,19,26,44] which has additionally been supported by the protective effect of several antioxidants in this experimental model [21-25].

**Table 1:** Quantitative analysis of 3-NT, DNP, catalase, Cu, Zn-SOD, and Mn-SOD immunostaining.

	Day 0	Day 2	Day 12
3-Nitrotyrosine	100 ± 11 (4)	392 ± 33 (4)*	113 ± 13 (4)
Dinitrophenol	100 ± 11 (4)	172.03 ± 9* (4)	106.81 ± 9 (4)
Catalase	100 ± 6 (5)	82 ± 6.7 (4)	107 ± 4.9 (4)
Cu, Zn-SOD	100 ± 3 (4)	90 ± 8 (4)	91 ± 7 (4)
Mn-SOD	100 ± 5 (3)	94 ± 6 (3)	110 ± 5 (3)

Data are expressed as percentage vs. day 0. *P < 0.001 vs. day 0.

Number of rats studied are in parentheses.

**Figure 6:**
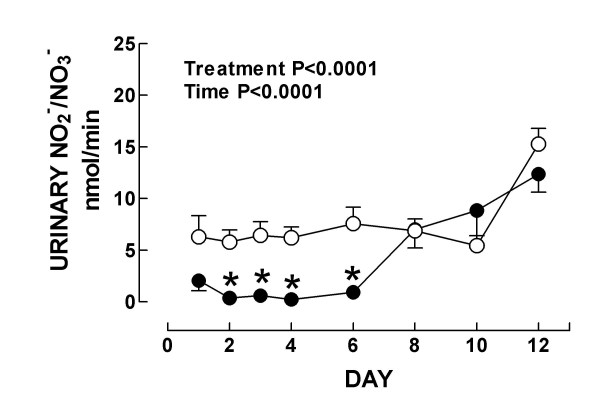
Urinary excretion of NO_2_^-^/NO_3_^- ^in control (○) and K_2_Cr_2_O_7 _(●)-treated rats. Data are mean ± SEM. *P < 0.05 vs. Control group. *n *= 4–10.

On the other hand, it was found that urinary excretion of NO_2_^-^/NO_3_^- ^decreased significantly in K_2_Cr_2_O_7_-treated rats on days 2–6 (Fig. 6). Urinary excretion of NO_2_^-^/NO_3_^- ^is considered as an index of NO production [37] and therefore these data may suggest that NO production is decreased in K_2_Cr_2_O_7_-treated rats. However, the strong nitrosative stress observed in our rats by 3-NT immunostaining, may suggest that the decrease in urinary NO_2_^-^/NO_3_^- ^excretion could be secondary to the NO consumption by its reaction with superoxide anion to generate peroxynitrite and other reactive nitrogen species involved in protein tyrosine nitration [46]. This is supported by the association between the time course alterations in 3-NT immunostaining (days 1–8) and the decrease in urinary NO_2_^-^/NO_3_^- ^excretion (days 2–6).

**Figure 7:**
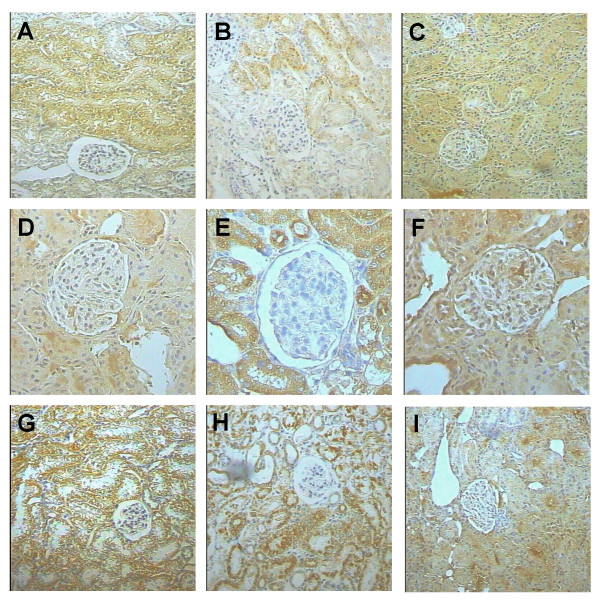
Representative immunohistochemical detection of CAT (first row), Cu, Zn-SOD (second row), and Mn-SOD (third row) during acute renal failure induced by K_2_Cr_2_O_7_. The study was performed in control rats (day 0) and on days 2 and 12 after a single injection of K_2_Cr_2_O_7 _(15 mg/Kg). (A) Normal kidney from control rats shows intense CAT immunoreactivity in the proximal and distal convoluted tubules. (B) In contrast, kidney cortex on day 2 shows evident decrease of CAT immunostaining. (C) Strong CAT immunoreactivity in the tubular epithelium is observed on day 12. (D) Normal kidney from control animals also showed strong Cu, Zn-SOD immunostaining in the cortex convoluted tubules. This strong Cu, Zn-SOD immunoreactivity is also observed on day 2 (E) and on day 12 (F). A similar pattern of Mn-SOD immunostaining is observed in normal kidney from control rat (G), and after 2 (H) and 12 (I) days of K_2_Cr_2_O_7 _injection. A-C and G-I 100X, D-F 400X.

**Figure 8:**
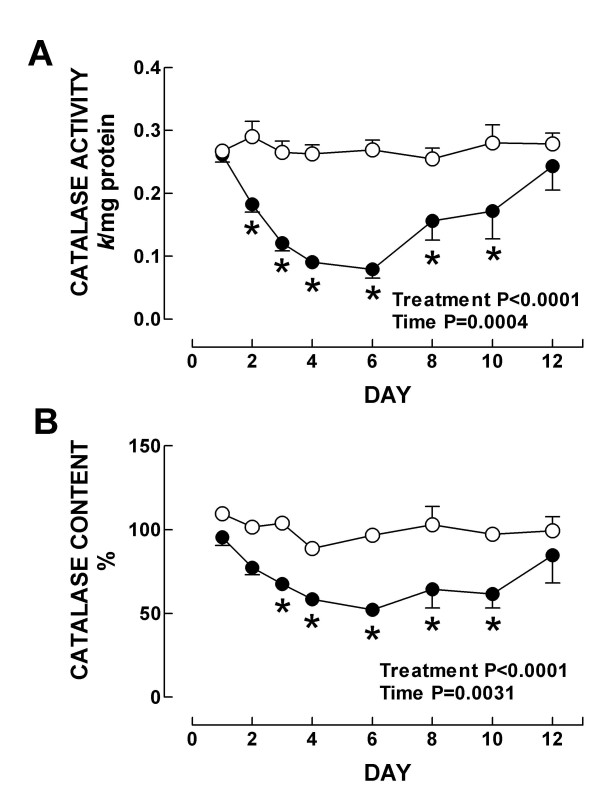
(A) Activity and (B) content of CAT in kidney from control (○) and K_2_Cr_2_O_7 _(●)-treated rats. Data are mean ± SEM. *P at least <0.05 vs. control group. *n *= 4–6.

**Figure 9:**
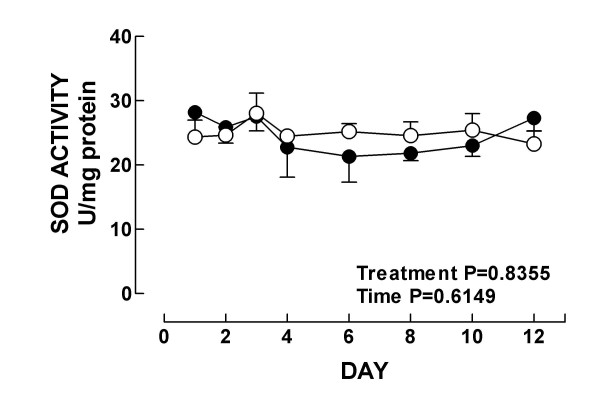
Total superoxide dismutase activity in kidney from control (○) and K_2_Cr_2_O_7 _(●)-treated rats. Data are mean ± SEM. *n *= 3–6.

**Figure 10:**
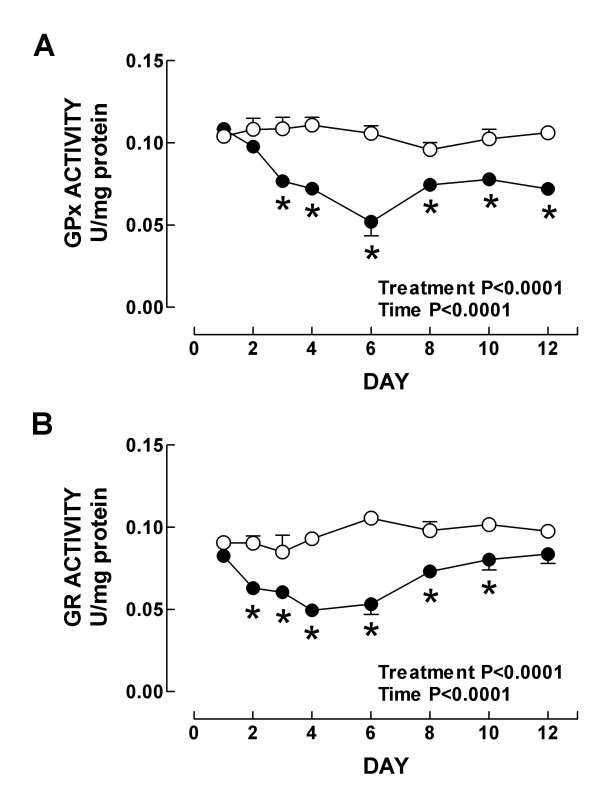
Activity of (A) glutathione peroxidase and (B) glutathione reductase in kidney from control (○) and K_2_Cr_2_O_7 _(●)-treated rats. Data are mean ± SEM. *P at least <0.05 vs. control group. *n *= 5–6.

Further we investigated the time response of the renal antioxidant enzymes in K_2_Cr_2_O_7_-treated rats. In control rats, strong CAT immunostaining was seen in the epithelial cells from proximal and distal convoluted tubules (Fig. 7A). From day 2 to day 10 after K_2_Cr_2_O_7 _administration, CAT immunostaining showed striking decrease, particularly in the epithelium from tubules with evident cellular damage (Fig. 7B and data not shown). Then, at day 12, when almost normal kidney histology was seen, strong CAT immunostaining was observed in a similar pattern than in the kidney from control animals (Fig. 7C). CAT activity (Fig. 8A) and content (Fig. 8B) decreased on days 2–10 and 3–10, respectively. In control animals the epithelial cells from the proximal and convoluted tubules showed strong immunoreactivity to Cu, Zn-SOD and Mn-SOD (Figs. 7D and 7G). This pattern of immunostaining did not show evident changes during the time course study (days 1–12) (Figs. 7E, 7F, 7H, 7I, and data not shown); even the damaged swollen epithelial tubular cells exhibited strong immunostaining to both SOD enzymes. These data are in agreement with kidney total SOD activity (Fig. 9) and with the protein content of Mn-SOD and Cu, Zn-SOD measured by Western blot which remained unchanged at all time points in K_2_Cr_2_O_7_-treated rats (data not shown). Finally, the activity of GPx and GR decreased on days 3–12 and on days 2–10, respectively (Fig. 10). It is very clear from the above data that there was a differential response of the antioxidant enzymes to K_2_Cr_2_O_7 _injection. Interestingly, no enzyme (SOD, CAT, GPx, and GR) was altered on day 1 when the kidney damage was very severe. Surprisingly, SOD activity and content remained essentially unchanged at all time points. This may be a consequence of the fact that both SOD enzymes were immunolocalized even in the damaged epithelial tubular cells. In contrast, the other enzymes decreased to reach their lowest values on day 6 returning then to basal values by day 12 with the exception of GPx which remained low. On days 2–6, there was some degree correlation between CAT, GPx, and GR and the markers of nephrotoxicity. However, from day 8 these enzymes remained low in spite of renal function and structure returned to control. Furthermore, the time course study suggests that the decrease in GPx, GR, and CAT activities may be secondary to the oxidative and nitrosative stress which are evident since day 1. In fact, it has been demonstrated that peroxynitrite impairs GPx activity [47] and superoxide anion inactivates GPx [48] and CAT [49]. Interestingly, GR and CAT remained low until day 10 and GPx remained low until day 12 in absence of oxidative and nitrosative stress. The reason why these enzymes remained low at the end of the study is not clear, however we may speculate that factors other than oxidative/nitrosative stress are involved in the diminution in these enzymes and/or proximal tubules have not reached yet the full capacity to synthesize these enzymes. Therefore, additional studies are required to explain why some antioxidant enzymes remained low on days 10–12 in absence of oxidative/nitrosative stress.

## Conclusion

The data show an association between oxidative/nitrosative stress and functional and structural renal damage induced by K_2_Cr_2_O_7 _in rats at different time points, but not between this damage and antioxidant enzymes.

## List of abbreviations used

ARF Acute renal failure

BUN Blood urea nitrogen

CAT Catalase

Cu, Zn-SOD Copper and zinc superoxide dismutase

DNP Dinitrophenol

DNPH 2,4-dinitrophenylhydrazine

ECL Enhanced chemiluminiscence

GPx Glutathione peroxidase

GR Glutathione reductase

H_2_O_2 _Hydrogen peroxide

K_2_Cr_2_O_7 _Potassium dichromate

Mn-SOD Manganese superoxide dismutase

NADPH Nicotinamide adenine dinucleotide phosphate

NAG N-acetyl-β-D-glucosaminidase

NBT Nitroblue tetrazolium

NO Nitric oxide

NO_2_^- ^Nitrate

NO_3_^- ^Nitrite

3-NT 3-Nitrotyrosine

ROS Reactive oxygen species

SEM Standard error of the mean

## Competing interests

The author(s) declare that they have no competing interests.

## Authors' contributions

JPCH conceived and coordinated the study and wrote and edited the manuscript. DB performed histological, immunohistochemical and statistical analyses. Coordinated the study and wrote the manuscript. ONMC performed Western blot analysis. RCC performed animal studies and collected samples. RHP performed the histological and immunohistochemical analyses. NAMR performed animal studies and collected samples. PDM measured antioxidant enzyme activity. MIS performed animal studies and collected samples. ET measured urinary excretion of NO_2_^-^/NO_3_^-^. LS and MEC measured chromium concentration. MEIR coordinated the study and edited the manuscript.

## Pre-publication history

The pre-publication history for this paper can be accessed here:



## Supplementary Material

Additional File 1 (A) Body weight, and (B) urinary volume in control (○) and K_2_Cr_2_O_7 _(●)-treated rats. Data are mean ± SEM. *P at least <0.01 vs. control group. *n *= 5–18. Click here for file
