# Supplementary material for: Time course study of oxidative and nitrosative stress and antioxidant enzymes in K2Cr2O7-induced nephrotoxicity
Source: BMC Nephrol. 2005 Apr 26;6:4. doi: 10.1186/1471-2369-6-4 (PMC1142323; doi:10.1186/1471-2369-6-4)
Supplement: Additional File 1 — (A) Body weight, and (B) urinary volume in control (○) and K2Cr2O7 (●)-treated rats. Data are mean ± SEM. *P at least <0.01 vs. control group. n = 5–18. [file 1471-2369-6-4-S1.doc]

**A**

**B**
